# 撤稿声明

**DOI:** 10.3779/j.issn.1009-3419.2010.11.19

**Published:** 2010-11-20

The editorial team of *Chinese Journal of Lung Cancer* (Zhongguo Fei Ai Za Zhi) recently confirmed that the manuscript written by Zhu ZT, *et al*, published in the Sep. 2010 issue of *Chinese Journal of Lung Cancer*, was a duplicate publication of their another paper in Zhong Liu Fang Zhi Yan Jiu.

Zhu Z, Yu Y, Wang K, Li E, Liu Y, Liu Y. Effect of Bufalin on Proliferation and Apoptosis of Human Non-small Cell Lung Cancer A549 Cell. Zhongguo Fei Ai Za Zhi, 2010, 13(9): 841-845. DOI: 10.3779/j.issn.1009-3419.2010.09.01

Yu Y, Liu Y, Wang K, Zhu Z, Xing D, Ha M. Effect of Bufalin on Inducing Cell Apoptosis and Its Mechanisms in Human Lung Adenoma Cells. Zhong Liu Fang Zhi Yan Jiu, 2010, 37(9): 1000-1003. DOI: 10.3971/j.issn.1000-8578.2010.09.007

These facts are apparent evidences of “duplicate publication” and the Editorial team decided to retract the paper from *Chinese Journal of Lung Cancer*.

The Editorial Office of *Chinese Journal of Lung Cancer*

Oct 19, 2010

《中国肺癌杂志》编辑部决定对[中国肺癌杂志, 2010, 13(9): 841-845]一文作撤稿处理。

题目：蟾蜍灵对人非小细胞肺癌A549细胞增殖与凋亡的影响

作者：朱志图 郁云龙^*^ 王锴 李恩泽 刘阳阳 刘云鹏

单位：121001 锦州，辽宁医学院附属第一医院肿瘤科

原因：此文与《肿瘤防治研究》 2010年37卷第9期通讯作者为朱志图的文章[郁云龙, 刘云鹏, 王锴, 朱志图^*^, 邢永达, 哈敏文. 肿瘤防治研究, 2010, 37(9): 1000-1003.]中大部分数据重复刊发，属于变相“重复发表”行为。

《中国肺癌杂志》编辑部

2010年10月19日

## Notes

https://www.ncbi.nlm.nih.gov/pmc/articles/PMC7969378

